# Antibody-drug conjugates in cancer therapy: applications and future advances

**DOI:** 10.3389/fimmu.2025.1516419

**Published:** 2025-05-21

**Authors:** Rou Long, Hanrong Zuo, Guiyang Tang, Chaohui Zhang, Xinru Yue, Jinsai Yang, Xinyu Luo, Yuqi Deng, Jieya Qiu, Jiale Li, Jianhong Zuo

**Affiliations:** ^1^ Institute of Translational Medicine, School of Basic Medical, Department of Special Medicine, Hengyang Medical College, University of South China, Hengyang, Hunan, China; ^2^ Hunan University of Chinese Medicine, Department of Clinical Medicine, Changsha, Hunan, China; ^3^ The Affiliated Nanhua Hospital of University of South China, Hengyang Medical School, University of South China, Hengyang, China; ^4^ Computer Institute, Hengyang Medical School, University of South China, Hengyang, Hunan, China; ^5^ The Third Affliated Hospital of University of South China, Hengyang Medical School, University of South China, Hengyang, China

**Keywords:** antibody-drug conjugates, ADCS, linker, probody-drug conjugate, dual-payload ADCs

## Abstract

Antibody-Drug Conjugates (ADCs) represent an emerging cancer therapeutic strategy and are becoming increasingly significant in the field of public health. With the evolution of precision oncology, the potential applications of ADCs are being realized more broadly. This review provides an overview of the fundamental molecular design of ADCs, examining how each component—antibody, linker, payload, and coupling chemistry—affects the physicochemical and biological properties of the final product. The paper also discusses novel ADC designs that are in preclinical and early clinical development stages as next-generation cancer therapies. These include bispecific ADCs, Probody-drug conjugate, immunostimulatory ADCs (ISACs), Degrader-Antibody Conjugates (DACs), and Dual-Payload ADCs. Their applications and potential future advancements in cancer therapy are also explored.

## 1 Introduction

Antibody-drug Conjugates (ADCs) have achieved remarkable clinical and commercial success, as evidenced by the recent approval and commercial performance of drugs like Fam-trastuzumab deruxtecan-nxki (Enhertu^®^), which has significantly impacted the industry (1). From 2000 to the end of 2023, a total of 13 ADCs have been approved by the U.S. Food and Drug Administration (FDA) for marketing (**Table 1**). Additionally, there are at least 100 ADCs in various stages of clinical trials (2) (**Table 2**).

**Table 1:** ADCs approved by the FDA as of 2023.

API	Brand name	Company	Linker	Cytotoxin	Target antigen	Indication	Approval Date
Gemtuzumab Ozogamicin	Mylotarg	Pfizer	Hydrazone	Calicheamicin	CD33	AML	2000-05-17
Brentuximab vedotin	Adcetris	Seagen,TakedaPharma	Dipeptide (VC)	MMAE	CD30	Hodgkin's Lymphoma;sALCL	2011-08-19
Trastuzumab emtansine	Kadcyla	Genentech	Non-cleavable (SMCC)	DM1	HER2	Breast Cancer	2013-02-22
Inotuzumab ozogamicin	Besponsa	Pfizer	Hydrazone	Calicheamicin	CD22	ALL	2017-08-17
Moxetumomab Pasudotox	Lumoxiti	AstraZeneca	Hydrazone	Pasudotoxtdfk	CD22	HCL	2018-9-13
Polatuzumab vedotin	Polivy	Genentech	Dipeptide (VC)	MMAE	CD79b	DLBCL	2019-06-10
Enfortumab vedotin	Padcev	Astellas Pharma US, Seagen	Dipeptide (VC)	MMAE	Nectin-4	Urinary bladder cancer	2019-12-18
Fam-trastuzumab deruxtecan	Enhertu	DaiichiSankyo	Non-cleavable (MC)	Deruxtecan	HER2	Metastatic breast cancer	2019-12-20
Sacituzumab govitecan	Trodelvy	Gilead Sciences	Acid-labile ester	SN-38	Tro2	Triple-negative breast cancer	2020-04-22
Belantamab mafodotin	Blenrep	GSK	Non-cleavable (MC)	MMAE	BCMA	Multiple myeloma	2020-08-05
Tisotumab vedotin	Tivdak	Seagen	Dipeptide (VC)	MMAE	TF	Recurrent or refractory metastatic castration-positive breast cancer	2021-09-20
Loncastuximab tesirine-lpyl	Zynlonta	ADC Therapeutics	Dipeptide (VA)	PBD	CD19	DLBCL	2021-04-23
Mirvetuximab soravtansine	Elahere	ImmunoGen	Sulfobenzoic acid (SPDB)	DM4	FRα	Ovarian cancer	2022-11-14

Gemtuzumab Ozogamicin: Initially approved by FDA in 2000, withdrawn in 2010, and re-approved in 2017.

**Table 2:** Selected ADCs in current clinical trials.

ADC Name	Company	Linker	Cytotoxin	Target Antigen	Indication	Phase
Glembatumumab vedotin	Seattle Genetics	Cleavable dipeptide	MMAE	gpNMB	Metastatic breast cancer; melanoma	II
PSMA	Progenics	Cleavable dipeptide	MMAE	PSMA	Prostate cancer	II
Pinatuzumab vedotin	Genentech	Cleavable dipeptide	MMAE	CD22	Diffuse large B-cell lymphoma	II
Telisotuzumab vedotin	Pierre Fabre	Cleavable dipeptide	MMAE	ABT-700	Advanced solid tumors cancer; non-small cell lung cancer	II
Ladiratuzumab vedotin SGN-LIV1A	Seattle Genetics	Cleavable dipeptide	MMAE	LIV-1	Breast cancer	II
Lorvotuzumab mertansine	ImmunoGen	Cleavable dipeptide	DM1	CD56	Leukemia	II
Coltuximab ravtansine	ImmunoGen	Cleavable dipeptide	DM4	CD19	Diffuse large B cell lymphoma; acute lymphocytic leukaemia	II
Indatuximab ravtansine	ImmunoGen	Cleavable dipeptide	DM4	CD138	Multiple myeloma	II
Anetumab ravtansine	Bayer Health Care	Cleavable dipeptide	DM4	Mesothelin	Mesotheliom;other solid tumors	II
SAR566658	Sanofi	Cleavable dipeptide	DM4	CA6	Triple-negative breast cancer	II
Naratuximab emtansine	ImmunoGen	Non-cleavable (SMCC)	DM1	CD37	Diffuse large B cell lymphoma; follicular lymphoma	II
AGS-16C3F	Astellas	Non-cleavable (MC)	ENPP3	ENPP3	Renal cell carcinoma	II
Rovalpituzumab tesirine	Sanofi	Dipeptide (VC)	PBD dimer	DLL3	Small-cell lung cancer	III
Mirvetuxima soravtansine	ImmunoGen	Cleavable dipeptide	DM4	FOLR1	Ovarian, endometrial, non-small cell lung cancer	III
Depatuxizumab mafodotin	AbbVie	Non-cleavable (MC)	MMAF	EGFR	Glioblastoma;other EGFR-positive tumors	III

The structure of oncology ADCs is characterized by the covalent binding of cytotoxic small molecule drugs to monoclonal antibodies (mAb) via a bifunctional linker in order to target binding to antigens overexpressed on the surface of tumor cells (3). This design effectively integrated biological components with small molecule drugs into a unified entity. However, this composite structure not only heightened technical complexity but also presented unique challenges in terms of chemical synthesis, manufacturing processes, and quality control (4).

In recent years, the emergence of novel constructs, including bispecific ADC, Probody-drug conjugate, immunostimulatory ADCs, Degrader-Antibody Conjugates(DACs)and Dual-Payload ADCs, has offered new avenues for addressing the aforementioned challenges (5). These innovative designs have been instrumental in enhancing tumor specificity and overcoming drug resistance. The market demand for ADCs is experiencing rapid growth, with an anticipated continuous expansion in the global market size. According to data from Frost & Sullivan, the global ADC market size has seen a swift increase from $1.6 billion in 2017 to $7.9 billion in 2024, with a Compound Annual Growth Rate (CAGR) of 37.3%. It is projected that by 2030, the global ADC market size will reach $64.7 billion, maintaining a CAGR of 30% (6–8).

Heterogeneity is a principal factor contributing to resistance to ADCs, which can lead to the development of resistance in certain tumor cells to ADC therapy, thereby limiting the efficacy of ADCs (9) (**Table 3**). Additionally, the tolerability of drugs is a substantial barrier in the advancement of ADCs, resulting in the market withdrawal of certain drugs attributed to their toxic profiles (9). Certain toxicities, including interstitial lung disease, pneumonia, and ocular toxicities, are associated with successful ADCs, despite their evident clinical effectiveness (10). Consequently, a comprehensive consideration of drug design and tumor characteristics is imperative to enhance the efficacy and safety profiles of ADCs.

**Table 3:** Mechanisms of tumor heterogeneity impacting ADCs efficacy.

Type of Heterogeneity	Impact on ADC Therapy	Representative Targets
Antigen expression heterogeneity	Subclones with low antigen expression evade ADC-mediated cytotoxicity, leading to acquired resistance	HER2, TROP2, CD22
Spatial heterogeneity	Differential antigen density between tumor core and periphery limits ADC penetration	EGFR, HER3
Temporal heterogeneity	Therapy-induced antigen phenotype shifts (e.g., HER2-positive → HER2-negative)	HER2, Nectin-4
Phenotypic plasticity	Epithelial-mesenchymal transition (EMT) mediated antigen downregulation	c-MET, CEACAM5

## 2 ADC structure analysis

The architecture of ADCs represents the central technological expertise within the pharmaceutical industry’s innovation pipeline, with a robust patent portfolio being crucial for competitive strength (11). Crafting an ADC requires a multifaceted approach that encompasses the antibody, linker, and small molecule drug, along with their synergistic integration (12). Identifying suitable antibodies initiates the ADC design sequence, and the linker and conjugation methodologies are paramount in shaping the drug’s therapeutic index. The small molecule drug within the ADC must demonstrate exceptional potency in eliminating cancer cells (13).

### 2.1 Antibodies in ADCs

In the realm of ADCs, antibodies serve as indispensable “vectors” for targeted drug delivery. They specifically bind to antigens on the surface of cancer cells, precisely delivering the payload to the tumor cells, thereby achieving the selective elimination of cancer cells (14). The structural and functional characteristics of antibodies fundamentally determine the pharmacokinetics and therapeutic efficacy of ADCs. Among *IgG* subtypes, *IgG1* is the dominant choice (85% of clinical-stage ADCs) due to its superior Fcγ receptor (FcγR) binding capacity and extended serum half-life (14–21 days) (15). This subtype not only enhances the antitumor immune response by activating innate immune cells such as natural killer (NK) cells and macrophages but also significantly reduces the immunogenicity of ADCs, thereby minimizing the formation of anti-drug antibodies (ADAs) (16).

To minimize off-target toxicity, the ideal target antigen should be highly expressed and tumor-specific, with little to no expression in healthy tissue cells (13). Common target antigens for marketed ADC drugs, such as CD22, CD33, CD30, and CD79, are highly expressed on the surface of cancer cells (13). Moreover, the stability of the antigen is crucial; a stable antigen reduces the likelihood of its detachment from target cells and subsequent binding to antibodies, thus avoiding the ineffective clearance of ADCs in the circulation (17).

An optimal ADC requires sufficient antigen expression on the surface of cancer cells (ideally greater than 10^5 per cell), with actual levels typically ranging from 5, 000 to 10^6 per cell (18). The payload of the drug is equally critical to its therapeutic efficacy. When selecting antibodies, high affinity and low immunogenicity are key considerations (19).

In targeted drug development, optimizing absorption efficiency and circulation time is essential for improving therapeutic efficacy. Enhancing internalization efficiency through antibody structure optimization or drug payload refinement is a key focus. However, excessively high antigen-antibody affinity may limit ADC penetration into deep tumor tissues due to the Binding Site Barrier (BSB) effect. To address this challenge, strategies such as adjusting antibody dose, reducing affinity, or using smaller antibodies (e.g., single-domain antibodies or scFv) can enhance ADC penetration (13, 20).

Emerging technologies, including chimeric antibody techniques, provide innovative solutions to these challenges by improving antibody properties such as stability and reduced immunogenicity. For instance, DS-8201a, an ADC targeting HER2, employs a tetrapeptide linker design that effectively masks the hydrophobicity of the drug molecule (21). This conjugation technology allows for the delivery of a high payload of hydrophobic drugs without compromising the pharmacokinetic properties of the antibody (21, 22). Additionally, site-specific conjugation can be achieved by incorporating specific peptide tags into the antibody structure, which are recognized and modified by enzymes such as transglutaminase, formylglycine-generating enzyme (FGE), or sortase (23, 24). This approach ensures efficient drug attachment to predefined sites on the antibody, preserving both its functional integrity and pharmacokinetic profile. Furthermore, chimeric antibody technology enables the design of smaller antibody constructs, such as single-domain antibodies or scFv, which can more effectively permeate tumor tissues, thereby improving drug delivery efficiency (25). Collectively, these advancements underscore the critical role of antibody engineering and conjugation technologies in enhancing therapeutic outcomes and advancing the field of precision medicine.

### 2.2 Payload in ADCs

Cytotoxic agents are the core payload in ADCs, commonly referred to as the “payload” (26). These payloads are transformed into potent cytotoxic drugs upon hydrolysis (26). In the development of anticancer ADCs, the payloads are primarily categorized into three classes: DNA-damaging agents, microtubule inhibitors, and topoisomerase inhibitors (27) (**Table 4**). When selecting these payloads, considerations beyond cytotoxicity include a comprehensive assessment of conjugation properties, solubility, and stability (20). The molecular structure of the drug should facilitate conjugation with linkers. Moreover, as ADCs are typically administered intravenously, the solubility and long-term stability in blood are particularly crucial, directly impacting the bioavailability and therapeutic efficacy of the drug (28).

**Table 4:** Representative small molecular cytotoxic payloads.

Category	Structure	Mechanism of Action
Microtubule inhibitors	MMAE	Inhibits microtubule polymerization, blocks mitosis
	MMAF	Inhibits microtubule polymerization
	DM1	Inhibits microtubule assembly (maytansinoid derivative)
	DM4	Inhibits microtubule assembly (maytansinoid derivative)
DNA damaging agents	Calicheamicin	Induces DNA double strand breaks via radical generation
	PBD	DNA minor groove crosslinking (pyrrolobenzodiazepine dimer)
	Duocarmycin	DNA alkylation
Topoisomerase inhibitors	SN-38	Topoisomerase I inhibitor
	DXd	Topoisomerase I inhibitor

Specifically, the solubility of the payload ensures that the ADC remains in solution during administration and circulation, preventing aggregation or precipitation that could lead to reduced bioavailability. The stability of the ADC in blood is also vital, as it prevents premature release of the payload, which could cause off-target effects and reduce the therapeutic index. Additionally, the bioavailability of ADCs administered intravenously is generally high, as this route bypasses the potential barriers of oral administration, such as first-pass metabolism and variable absorption (29). However, factors such as the drug-to-antibody ratio (DAR), linker chemistry, and the molecular weight of the ADC can influence its pharmacokinetics and, consequently, its bioavailability (30). For instance, a high DAR may lead to increased hydrophobicity and potential aggregation, which could affect the stability and clearance of the ADC in the bloodstream (29, 30). Therefore, optimizing these factors is essential to maximize the therapeutic potential of ADCs.

The DAR is a key quality attribute of ADCs, representing the average number of small molecule drugs conjugated per antibody (31). The DAR value significantly influences the pharmacokinetics, efficacy, and toxicity profile of ADCs (32). Lower DAR values (eg., 2 to 4) contribute to more stable drug distribution and prolonged therapeutic effects, while higher DAR values may lead to excessive drug accumulation in healthy tissues, triggering stronger toxic reactions (33). The ideal DAR must balance the anticancer potency of the ADC with its safety.

Due to the lysosomal barrier and the complexity of the tumor microenvironment, the number of cytotoxic payloads that can effectively reach their target sites is limited. However, payloads with low IC50 values are often considered ideal candidates for efficient ADCs. For DNA-damaging agents, IC50 values typically range in the picomolar range, while microtubule inhibitors tend to exhibit potency in the nanomolar range (34). For example, the IC50 values of Duocarmycin and Pyrrolobenzodiazepines are reported to be 1-10 pM and 0.1-1 pM, respectively, demonstrating potent tumor cell-killing effects (35). In addition, Calicheamicins and Exatecans, two well-established DNA-damaging agents, have IC50 values in the range of 0.1-1 nM and 1-10 nM, respectively, and show significant therapeutic potential (35). However, despite the impressive preclinical efficacy of these highly potent agents, their clinical development must proceed with caution, as some picomolar agents have been discontinued during clinical trials due to severe adverse effects (4).

The development of ADCs is contingent upon the selection of highly potent payloads, precise control of DAR, and the application of site-specific conjugation technologies. These critical factors collectively dictate the therapeutic efficacy and safety profiles of ADCs. With ongoing technological advancements, the field of ADCs is poised to unveil a new generation of highly effective, low-toxicity drugs, offering renewed hope for cancer therapeutics.

### 2.3 Linkers in ADCs

Linkers play an indispensable role in ADCs, covalently binding cytotoxic payloads to mAbs to ensure drug stability in the bloodstream and effective release upon reaching tumor cells (36). The design of linkers must balance stability with drug release efficiency to maximize therapeutic efficacy and minimize side effects.

Linkers can be categorized into two main classes: cleavable (degradable) and non-cleavable (stable) (37). Cleavable linkers exploit the unique environmental conditions of tumor cells, such as low pH, proteolytic activity, or a reductive environment, to trigger drug release (38). These linkers are stable under normal physiological conditions but undergo cleavage in the acidic or reductive environment specific to tumor cells, thereby releasing the cytotoxic drug (39). Common examples of cleavable linkers include acid-sensitive, protease-sensitive, and glutathione-sensitive linkers. For instance, hydrazone bonds, disulfide bonds, and peptide linkers are typical cleavable linkers (40). While these linkers effectively release drugs, they may possess certain instabilities, leading to premature drug release before reaching tumor cells and increasing the risk of off-target toxicity (37). The bystander effect, a phenomenon where the cytotoxic payload released from ADCs can affect neighboring tumor cells, even those that do not express the target antigen, thereby enhancing the overall therapeutic efficacy, is particularly relevant here (41). This effect is more effectively harnessed when the linker design ensures optimal drug release within the tumor microenvironment.

Non-cleavable linkers (stable linkers) are resistant to hydrolysis in the bloodstream and typically require degradation within the lysosomes of cells, thus having a longer half-life in the bloodstream and reducing the risk of off-target toxicity (41). Common stable linkers include thioether.

To overcome these limitations, scientists have developed various innovative approaches. For example, the use of fully alkylated interchain disulfide bond technology can enhance the stability of ADCs and reduce unnecessary drug release (42). Additionally, the THIOMAB80 technology introduces specific amino acid residues into antibodies through engineering methods to optimize linker conjugation and stability (43, 45). Non-natural reactive amino acid conjugation techniques and engineered enzyme-mediated conjugation are also employed to achieve precise conjugation, thereby enhancing the targeting and efficacy of ADCs (28).

In the development of ADCs, selecting the appropriate linker remains a critical challenge. The ideal linker should remain stable in the bloodstream while rapidly and effectively releasing the payload within tumor cells (18). As ADC technology continues to advance, the design and optimization of linkers will continue to drive the development of new ADC drugs, thereby improving therapeutic outcomes and reducing adverse effects.

## 3 Mechanism of action of ADCs

ADCs represent a transformative therapeutic modality in oncology, integrating the precision of mAbs, the controlled release of cytotoxic payloads, and the potency of chemotherapeutic agents to achieve selective tumor eradication. The mAb component, characterized by its nanomolar binding affinity for tumor-specific antigens, facilitates prolonged systemic circulation and targeted tumor accumulation while minimizing off-target effects on healthy tissues (28, 37, 46). Upon binding to tumor-specific antigens, the ADC-antigen complex is internalized via clathrin or caveolin-mediated endocytosis, forming early endosomes. These endosomes subsequently acidify and mature into lysosomes, where the acidic environment and proteolytic enzymes trigger payload release (47–49).

**Figure 1:**
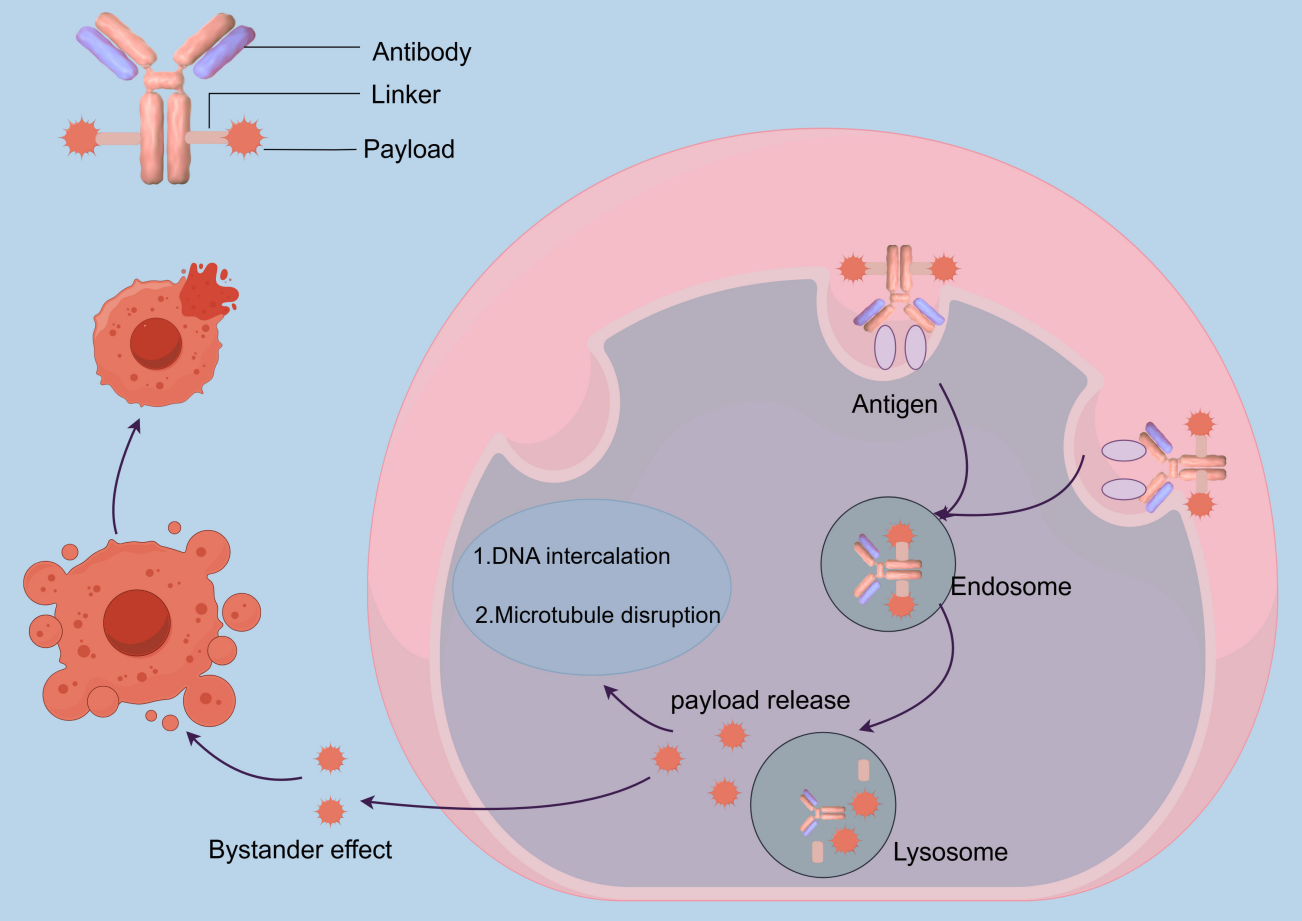
Illustrates the mechanism of action of ADCs.

The released cytotoxic payload exerts its antitumor effects through two primary mechanisms. Microtubule disrupting agents, such as auristatins, bind to β-tubulin, destabilizing microtubule dynamics and arresting mitosis, ultimately leading to mitotic catastrophe. Conversely, DNA damaging-agents, such as calicheamicin, induce double strand breaks or DNA crosslinking, activating p53-dependent apoptosis through the intrinsic mitochondrial pathway (28). A distinctive feature of ADCs is the bystander effect, wherein hydrophobic payloads diffuse into adjacent antigen - negative tumor cells, thereby overcoming spatial heterogeneity in antigen expression and enhancing therapeutic efficacy (50, 51) (**Figure 1**).

The coordinated mechanism of action encompassing antigen recognition, lysosomal processing, and cross-cell cytotoxicity results in robust tumor eradication with reduced systemic exposure. This integration of biological targeting and chemical precision positions ADCs as a pivotal advancement in precision oncology, offering a paradigm shift in the treatment of heterogeneous tumors while maintaining a favorable therapeutic index (52).

## 4 Challenges facing ADCs

ADCs have emerged as a transformative modality in oncology therapeutics. However, their clinical translation necessitates resolution of multifaceted challenges across pharmacokinetics, structural design, and translational processes.

### 4.1 Pharmacokinetic complexity

The dynamic pharmacokinetic (PK) behavior of ADCs presents significant clinical hurdles. *In vivo* transformations generate heterogeneous species including intact ADCs, naked antibodies, and free cytotoxic payloads, with temporal variations in their relative proportions. This complexity impedes the establishment of reliable dose-response relationships and complicates PK modeling, ultimately affecting both therapeutic predictability and drug development efficiency (53).

### 4.2 Payload release control and toxicity

Premature payload dissociation remains a critical safety bottleneck. Covalent linkage instability, particularly in conventional lysine/cysteine conjugation systems, leads to systemic release of cytotoxic agents. Such off-target release correlates strongly with dose-limiting hematotoxicity and organ damage (54). Recent studies reveal that approximately 40% of ADC-related adverse events originate from suboptimal linker stability (55), underscoring the urgent need for spatiotemporally controlled release mechanisms.

### 4.3 Resistance mechanisms

ADC resistance develops through three primary pathways: (1) antigen-mediated mechanisms including target downregulation and epitope masking; (2) intracellular processing defects such as impaired lysosomal acidification; and (3) payload-specific adaptations like drug efflux pump activation. While combinatorial regimens and next-generation payloads show preclinical promise, their clinical validation rates remain below 30% in phase II trials (53, 56), highlighting fundamental gaps in resistance biology understanding.

### 4.4 Target antigen heterogeneity

Temporal-spatial heterogeneity in tumor antigen expression creates dual therapeutic challenges. First, inter-patient variability (e.g., 2-5 log differences in HER2 expression across breast cancer subtypes) requires population-level stratification. Second, intra-tumor clonal evolution demands real-time adaptation of targeting strategies. To address this multidimensional heterogeneity, emerging approaches combine two critical parameters: quantitative antigen density thresholds (>5, 000 receptors/cell to ensure effective internalization), and bystander-effect optimization. This dual strategy enhances therapeutic efficacy through complementary mechanisms - while antigen thresholds guarantee sufficient drug uptake in high-expression cells, the bystander effect extends cytotoxicity to neighboring cancer cells with lower antigen expression (56).

### 4.5 Structural optimization challenges

Two critical parameters govern ADC efficacy-toxicity balance: The DAR and conjugation homogeneity. While DAR values exceeding 4 enhance payload delivery, they concurrently increase plasma clearance rates by 60-80% through accelerated macrophage uptake (53). Parallel improvements in site-specific conjugation technologies (e.g., engineered cysteine residues achieving >95% homogeneity) and stable linker chemistries (e.g., sulfatase-cleavable systems) are essential for next-generation constructs (55).

### 4.6 Manufacturing and clinical translation barriers

The intricate tripartite structure of ADCs imposes stringent Chemistry, Manufacturing, and Controls (CMC) requirements. Key production challenges include maintaining conjugation efficiency within ±5% batch variability and ensuring payload stability during lyophilization processes. These technical hurdles contribute to development costs exceeding $500 million per approved ADC, with 67% of clinical-stage candidates failing due to inadequate therapeutic indices (53, 54, 57). CMC directly addresses therapeutic index challenges by optimizing critical quality attributes including DAR uniformity, minimizing premature payload release (reducing off-target toxicity), and maintaining antibody specificity (preserving target engagement) (53). Through rigorous process controls in conjugation chemistry, formulation stabilization strategies, and advanced analytics for characterization, CMC ensures product consistency that enhances the safety efficacy balance required for clinical success (57).

### 4.7 Target homogenization and market viability

Current ADC development exhibits concerning target redundancy, with 43% of clinical candidates targeting HER2 or TROP2 (12). This concentration creates therapeutic duplication (e.g., 8 anti-HER2 ADCs in phase III trials) while neglecting emerging targets like CLDN6 and PTK7. Such market saturation risks diminishing commercial returns and stifling innovation in target discovery.

Innovative engineering strategies are reshaping ADC development trajectories. Bispecific platforms (e.g., HER2xCD3 dual-targeting ADCs) demonstrate 3.2-fold improved tumor selectivity in primate models (5), while conditionally activated prodrug linkers reduce systemic toxicity by 89%. Concurrent advances in companion diagnostics, particularly circulating tumor DNA-based antigen monitoring, may enable real-time therapeutic adaptation, positioning ADCs as precision oncology cornerstones.

## 5 Future directions in ADCs

### 5.1 Improving ADC domains

#### 5.1.1 Antibody

The selection of antibodies is crucial for the development and efficacy of ADCs. Humanized and fully human mAbs have become the most commonly used types in ADC development due to their excellent properties, such as low immunogenicity, long half-life, and potent immune response capabilities (58, 59). However, the success of ADCs also depends on the careful selection of antigens that are highly expressed in tumor cells and minimally expressed in normal tissues. Many successful ADC targets, such as HER2 and TROP2, are also expressed in certain normal tissues, which can lead to target-dependent and off-target toxicity reactions, potentially resulting in the suspension or premature termination of clinical trials (60). To mitigate these challenges, strategic antibody engineering approaches such as truncation (removal of non-essential antibody domains) and clipping (controlled proteolytic processing of specific regions) are increasingly employed to refine target specificity. For instance, Fc domain truncation eliminates Fc-mediated interactions with immune cells in normal tissues, thereby reducing nonspecific uptake and cytokine release syndrome risks. Conversely, Fab region clipping can optimize antigen-binding fragment geometry for enhanced epitope discrimination between tumor-associated antigens and their physiological counterparts (61). These structural modifications work synergistically with glycosylation engineering and other post translational optimizations to create “tumor-selective” antibody architectures that minimize off target binding while maintaining payload delivery precision to malignant cells (16).

To further enhance the therapeutic efficacy of antibodies, researchers have been modifying the Fc region of antibodies to augment their cytotoxic activity (19). These modifications can be categorized into two main approaches: structural engineering of the Fc region and glycan remodeling. Structural engineering includes site-directed mutagenesis and asymmetrical engineering (62). Site-directed mutagenesis, such as the S239D/I332E mutations in the Fc region, has been shown to significantly improve binding to FcγR IIIa, leading to enhanced ADCC (63). These mutations have been incorporated into anti-CD19/CD40 antibodies, demonstrating improved treatment efficacy in preclinical and clinical studies. Asymmetrical engineering of the Fc region to create heterodimers of different heavy chains has also been shown to yield more stable antibodies with improved ADCC functionality, and this approach has been used in the development of bispecific antibodies for cancer therapy (64–66).

Glycan remodeling involves techniques such as afucosylation and oxidation-based glycan remodeling. Afucosylation, which removes fucose from the Fc region, has been demonstrated to significantly enhance binding to FcγR IIIa, leading to higher levels of ADCC both *in vitro* and *in vivo* (63). The POTELLIGENT^®^ technology, licensed by Kyowa Hakko Kirin Co., uses FUT8 knockout CHO cells to generate afucosylated antibodies (67). Examples of afucosylated antibodies include mogamulizumab (anti-CCR4 antibody), which has shown superior efficacy in clinical trials and is now approved for the treatment of certain blood cancers (68). Oxidation-based glycan remodeling, such as periodate oxidation, can generate aldehyde groups on the Fc region, enabling site-specific conjugation of cytotoxic payloads (69). This technique has been used in the development of ADCs to enhance their therapeutic potential.

These modifications not only enhance the cytotoxic activity of antibodies but also improve their therapeutic potential in cancer treatment.

#### 5.1.2 Payload

In recent years, the introduction of novel payloads such as immunomodulators and targeted protein degraders has opened new directions for the development of ADCs.

Immunomodulators, including certain immune checkpoint inhibitors (such as PD-1 and CTLA-4 inhibitors), have been incorporated into ADCs designs to enhance immune responses within the tumor microenvironment (70, 71). For instance, the immunosuppressive agent Voclosporin (Lupkynis) has received FDA approval for the treatment of systemic lupus erythematosus. Voclosporin modulates immune responses by inhibiting calcineurin to reduce T-cell activation (72, 73).

Targeted protein degraders, including molecular glue and proteolysis-targeting chimeras (PROTACs), are a class of drugs that induce the degradation of target proteins by recruiting the E3 ubiquitin ligase system (74). For example, the molecular glue BI-3802, targeting BCL6, has shown potential in treating B-cell related cancers by inducing BCL6 degradation (71). Furthermore, CelMoDs, a class of IKZF1/3 degraders developed by Bristol-Myers Squibb (such as CC-92480, CC-220, and CC-99282), have entered clinical studies for the treatment of relapsed or refractory multiple myeloma (RRMM) (75).

In the research and application of ADCs, the hydrophobicity of the toxin is a critical factor affecting its efficacy and side effects and is closely related to the “bystander effect” (46). Hydrophobic toxin molecules, such as MMAE, can diffuse passively into neighboring tumor cells, producing a bystander effect that enhances the broad cytotoxic effect of ADCs on antigen-expression heterogeneous tumors (76). However, when toxin molecules are overly hydrophobic, they may aggregate *in vivo*, be phagocytosed, or undergo nonspecific binding, increasing off-target toxicity (76).

To mitigate the side effects of highly hydrophobic toxins, researchers have proposed a strategy of introducing hydrophobicity-masking groups on toxin molecules, such as polyethylene glycol (PEG) or polyglutamic acid (77). These masking groups increase the solubility of ADCs and reduce nonspecific interactions with normal tissues, thereby alleviating side effects caused by highly hydrophobic toxins, such as aggregate formation, rapid clearance, and potential immunogenicity (78). This strategy allows ADCs to maintain favorable pharmacokinetic properties and lower off-target toxicity even at high DAR, thereby further enhancing therapeutic efficacy (79, 80).

#### 5.1.3 Linkers

The design and construction of linkers are vital for enhancing the efficacy and safety of ADCs. Traditional random conjugation methods often result in product heterogeneity, which can impact the consistency of pharmacokinetics and efficacy, and may lead to drug aggregation, off target toxicity, and structural instability (50, 51, 53). To address these challenges, site-specific conjugation has emerged as a revolutionary approach in bioconjugation chemistry (44). This method enhances therapeutic consistency and optimizes structure-function relationships by precisely targeting reactive sites on biomolecules such as antibodies and proteins (44, 57, 81).

Techniques employed for site specific conjugation include the use of engineered cysteine residues, unnatural amino acids, enzymatic methods, or affinity guided peptides (44). These techniques achieve positional control, minimizing product heterogeneity and preserving critical epitope-binding regions. For instance, site specific ADCs using engineered cysteines have demonstrated superior pharmacokinetic profiles and reduced aggregation propensity compared to lysine-conjugated counterparts (53). Similarly, the incorporation of non-natural amino acids enables bioorthogonal click chemistry conjugation, which has been validated in tumor-targeting nanocarriers (82).

Recent innovations in enzymatic conjugation have further enhanced specificity through sequence recognition motifs. For example, microbial transglutaminase (mTG) mediated conjugation enables site-specific drug attachment at glutamine residues without antibody sequence modification, achieving DAR homogeneity and reduced aggregation (DAR 2-4 with <10% variability) (83). This approach, combined with Diels Alder chemistry, forms carbon-carbon bonds between linkers and antibodies, significantly improving *in vivo* stability compared to traditional maleimide-thiol conjugations (84). Moreover, dual conjugation strategies (e.g., attaching both cytotoxic drugs and immune modulators to a single antibody) are emerging as a frontier, leveraging orthogonal conjugation sites (e.g., engineered cysteines and glycan-modified residues) to broaden therapeutic applications (85, 86). This level of precision is crucial for next generation ADCs and bispecific antibodies, where payload stoichiometry directly impacts the therapeutic index and off-target toxicity (5).

In-depth exploration and optimization of linker design and construction strategies are crucial for improving drug efficacy and minimizing toxicity. Research teams have developed novel linker technologies, such as dendrimeric dimeric linker technology, to create ADCs with high DAR (87). This technology reduces non-specific interactions between the antibody and the toxin, enhances drug stability, and improves drug delivery efficiency, ensuring more precise delivery of the toxin to the tumor target (88, 89).

Additionally, researchers have developed lysosomal enzyme-degradable linkers, such as ValCit-PABC and AlaAlaAsn-PABC-MMAE (90). These linkers are designed to release the toxin specifically within the lysosomes of tumor cells, thereby reducing drug toxicity. However, challenges such as premature cleavage in mouse plasma due to Ces1C enzyme degradation have been addressed by introducing acidic amino acids at specific positions to enhance linker stability (90).

In summary, precise linker design strategies, including selecting appropriate linker types, optimizing stability and adaptability, and improving *in vivo* stability, are driving ADCs research toward enhanced efficacy, reduced toxicity, and increased specificity (40). As the diversity of tumor types, targets, and drug types continues to expand, customized linker design strategies are becoming increasingly important for optimizing ADCs efficacy and safety (40). Research is also focusing on linker stability within the tumor microenvironment, particularly in acidic conditions, offering new hope and potential solutions for cancer therapy (12).

### 5.2 Novel ADCs

#### 5.2.1 Biepitopic and bispecific ADCs

To overcome resistance to single-target ADCs, researchers are investigating novel approaches. Biepitopic antibodies, which attach to two separate epitopes on the same antigen, have emerged as an effective strategy to surpass the limitations of single-target ADCs (91). This improved stability profile and tumor targeting efficiency directly translate to enhanced therapeutic efficacy. Mechanistically, the preserved structural integrity of site specific conjugates minimizes premature payload release in circulation, thereby maintaining cytotoxic concentrations at tumor sites to prevent subtherapeutic exposure that drives resistance (92). Emerging clinical evidence further demonstrates that ADCs with DAR homogenization achieved through site-specific conjugation exhibit significantly reduced multidrug resistance protein 1 (MDR1)-mediated drug efflux compared to their heterogeneous counterparts (93).

Biepitopic ADCs offer a new method in cancer treatment by allowing a single antibody to engage two different epitopes on an antigen present on cancer cells. This configuration boosts binding affinity, especially in cancer cells with low HER2 levels, and increases drug delivery efficiency (94) (**Table 5**). For example, biepitopic ADCs targeting two distinct HER2 epitopes can form receptor-antibody complexes on the cell surface, facilitating endocytosis and lysosomal trafficking while decreasing antigen expression (47, 95–97).

**Table 5:** Bispecific ADCs at AACR 2024.

Target pair	Asset lD	Company	Format	Cytotoxin	Additional tech	Stage
EGFR×B7H3	IBI3001	Innovent	1+1	Topo	Fc silentSynaffi×SS	Preclinical
Nectin4×TROP2	VBC103	VelaVigo	2+1	Topo	/	Preclinical
EGFR×MET	PRO1286	Profound	1+1	Topo	/	Preclinical
HER2×TROP2	BI0-201	BiOneCure	2+2 (Fab/ScFv)	Topo	/	Discovery
EGFR× HER3	BCG019	Biocytogen	1+1	Topo	Common LC	Discovery
HER3×MUC1	DM002	Biocytogen	1+1	Topo	Common LC	Preclinical
EGFR×HER3	PM1300	Biotheus	1+1	Topo	/	Discovery
HER3×MET	BCG022	Biocytogen	1+1	Topo	/	Discovery
PTK7×TROP2	BCG033	Biocytogen	2+2	Topo	Common LC	Discovery
Axl×PD-L1	CPBT0976	Celon	2+2(VHH)	MMAE	/	Discovery
5T4×MUC1	BCG016	Biocytogen	1+1	MMAE	Common LC	Discovery
EGFR×PTK7	BCG017	Biocytogen	1+1	MMAE	Common LC	Discovery
FRa×MUC1	BCG023	Biocytogen	1+1	MMAE	Common LC	Discovery
EGFR×TROP2	DXC024	Hangzhou	1+1 (hybrid)	Tubulysin	/	Discovery
EGFR×MUC1	DXC025	Hangzhou	1+1(hybrid)	Tubulysin	ConjuAll SS	Discovery
CD20×CD22	LCB36	LigaChem	1+1	Masked PBD	ConjuAll SS	Preclinical
EGFR×MET	VBC101	VelaVigo	2(bip) +1	MMAE or Topo	Common LC	Discovery

To assess the performance of biepitopic ADCs, scientists have created novel tetravalent bispecific antibodies (BsAbs) targeting cMet/EGFR and cMet/HER2 (98). These BsAbs link antibodies that induce rapid internalization and degradation of Met with single-chain variable fragments targeting EGFR or HER2 (99, 100). Furthermore, combining HER2 with other targets like B7-H3 and B7-H4 holds promise for broader therapeutic applications (101). For instance, a HER2×CD63 bispecific ADCs demonstrated increased cytotoxicity in HER2-positive tumors, highlighting the potential for more precise targeting.

Addressing the resistance challenges of single-target ADCs, scientists have turned to bispecific antibodies as a primary strategy. Their ability to target two antigens not only improves therapeutic outcomes but also diminishes the risk of resistance.

SI-B001, a bispecific antibody against EGFR and HER3, has been linked with a novel AC linker and the topoisomerase I inhibitor ED04 to form the BsADC BL-B01D1, which has a DAR of approximately 8. This conjugate improves targeting precision and safety (102). BL-B01D1 specifically targets EGFR-dependent tumors and mitigates HER3-related resistance through dual-target crosslinking and internalization (103). Phase I trials, particularly in patients with EGFR-mutated non-small cell lung cancer (NSCLC), reported a 63.2% objective response rate (ORR), demonstrating its effectiveness against multidrug resistance (104).

Currently, BsADC development focuses on targets like HER2, cMet, and EGFR, with increasing interest in other targets such as B7-H3 and B7-H4. Additionally, the bispecific approach shows potential for targeting antigens with low expression or poor internalization (105). Advances in non-*IgG*-like bispecific antibodies are addressing half-life challenges, thereby enhancing cancer therapy precision (106). Moreover, the creation of trispecific and multifunctional antibodies offers promising strategies to overcome receptor redundancy and tumor heterogeneity, paving the way for personalized treatments (18).

#### 5.2.2 Probody-drug conjugate

Traditional ADCs targeting receptors are often expressed not only on cancer cells but also on certain non-malignant tissues, which is commonly associated with inescapable targeted non-tumoral toxicity, leading to dose reduction or treatment interruption (5). To address this issue, a new class of ADC designs featuring conditionally active antibodies, commonly referred to as Probodies, has been developed (5).

This concept is inspired by small molecule prodrugs, where the toxin is delivered to the body in an inactive form and is converted to its active form in the circulatory system or specific organs, thereby improving drug stability and specificity (107).

Probodies are engineered by fusing a self-masking moiety to the N-terminus of an *IgG* molecule through a cleavable spacer or are designed to exhibit reduced binding affinity under pH-dependent conditions (5, 107). Upon reaching the tumor microenvironment, the masking moiety of Probodies is either removed or undergoes a conformational change due to the presence of proteases and acidic conditions, thereby restoring the antibody’s affinity and triggering the release of the cytotoxic payload (107).

**Figure 2:**
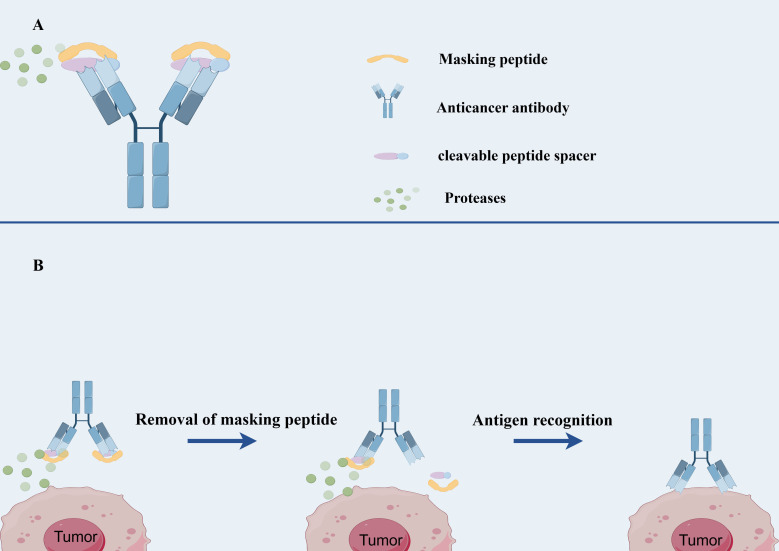
The structure and mechanism of Probody-drug conjugate. **(A)** The structure of Probody-drug conjugate. **(B)** the mechanism of action of Probody-drug conjugate.

Probody technology encompasses a class of recombinant antibody-based therapeutics that are activated through proteolytic cleavage. These constructs consist of three principal components: a monoclonal antibody with anticancer activity or a fragment of its variable region, a masking peptide attached to the N-terminus of the light chain that obscures the antibody’s Fab region from antigen binding, and a peptide linker (spacer) susceptible to enzymatic cleavage, which connects the peptide to the antibody (107, 108) (**Figure 2**). Probody therapeutics can be produced using conventional recombinant antibody manufacturing techniques. The mechanism of action of Probody relies on the differential protease activity between normal and tumor tissues, thereby confining the drug’s activity predominantly to the tumor microenvironment and minimizing activity in normal tissues (109). In normal tissues, the Probody maintains its intact structure, and due to the presence of the masking peptide, the antibody is unable to bind antigens, resulting in an extended half-life (110). Upon reaching the tumor microenvironment, proteases cleave the linker, releasing the masking peptide and exposing the antibody’s Fab region for antigen binding (109) (**Figure 2**).

A significant advantage of Probody technology is its theoretical applicability to a broad range of antibody-based drugs (111). This technology has been successfully applied to various antibody types for therapeutic purposes, including immunomodulatory factors and immune checkpoint inhibitors (e.g., anti-PD-L1), ADCs (anti-CD71, anti-CD166, anti-EGFR), and T-cell bispecific antibodies (anti-EGFR-CD3) (112, 113).

Early research identified a peptide sequence (LSGRSDNH) susceptible to cleavage by multiple proteases, which exhibits low activity in non-malignant tissues but is upregulated in the microenvironment of various human cancers (114). Preclinical animal studies demonstrated a roughly tenfold increase in therapeutic index upon the introduction of a masking group. Building on this innovation, researchers have developed several pioneering Probody-drug conjugates, currently in the preclinical phase, including CX-2051, praluzatamab ravtansine (formerly CX-2009), NCT03149549, NCT04596150, and CX-2029 (108, 112, 113, 115).

Furthermore, the tumor microenvironment (TME) typically exhibits acidity (pH 6.0-6.8), in contrast to the neutral pH of normal tissues (7.3-7.4), providing a basis for the conditional activation of ADCs (5). Consequently, the introduction of weakly basic histidine into the antibody-binding region has become a common strategy to achieve pH-dependent activation. A variety of pH-dependent ADCs have been developed, targeting EGFR, HER2, AXL175, and ROR2, among others (116, 117).

For instance, the EGFR-targeting Probody-drug conjugate HTI-1511, based on the microtubule inhibitory agent MMAE, has shown promising preclinical data (118). This conjugate exhibits over ten times greater binding affinity to EGFR at pH 6.0-6.5 compared to pH 7.4 and maintains binding capability comparable to cetuximab in EGFR-expressing A431 xenografts (118). Moreover, in cetuximab-resistant PDX models and mouse models harboring KRAS or BRAF mutations, HTI-1511 significantly inhibited and even reversed tumor growth. In cynomolgus monkey studies, HTI-1511 demonstrated good tolerability at doses up to 8 mg/kg, suggesting favorable clinical safety (118).

However, since 2018, the clinical development of HTI-1511 appears to have stalled, possibly due to potential competition in the EGFR ADC field, technical challenges in scaling up production, and shifts in corporate strategic focus (5).

As an example of a novel Probody-drug conjugate responsive to the TME, researchers have developed a pH-dependent Probody-drug conjugate based on a mechanism known as protein-associated chemical switch (PAC). The complementarity-determining regions of these Probody-drug conjugates are designed to interact with abundant ions, including sodium chloride, bicarbonate, and hydrogen sulfide (119). At a pH of approximately 7.4, the negatively charged forms of these molecules exist at sufficient concentrations to inhibit antigen binding through interactions with positively charged complementarity-determining regions (119). However, in the more acidic TME, the concentration of these ions decreases, enabling ion concentration-dependent activation of target binding.

#### 5.2.3 Immune receptor agonists

Over the past decade, cancer immunotherapy has made significant strides, sparking renewed interest in this field (5). Damage-associated molecular patterns (DAMPs) released by tumor cells can trigger innate immune activation. In this context, immune adjuvant molecules, by interacting with pattern recognition receptors (PRRs), have become a focal point in cancer drug development (5). The combination of ADCs with PRR agonists is regarded as a promising strategy for locally activating the innate immune system.

Compared to traditional ADCs, immunostimulatory ADCs possess several potential advantages. Firstly, they can target a variety of tumor-associated DAMPs, thereby promoting antitumor immune responses (120). Secondly, immunostimulatory ADCs not only activate antigen-presenting cells (APCs) but may also stimulate other tumor-infiltrating immune cells, such as T cells. Additionally, they can elicit immune memory effects, providing durable antitumor action and reducing the risk of recurrence (120).

**Figure 3:**
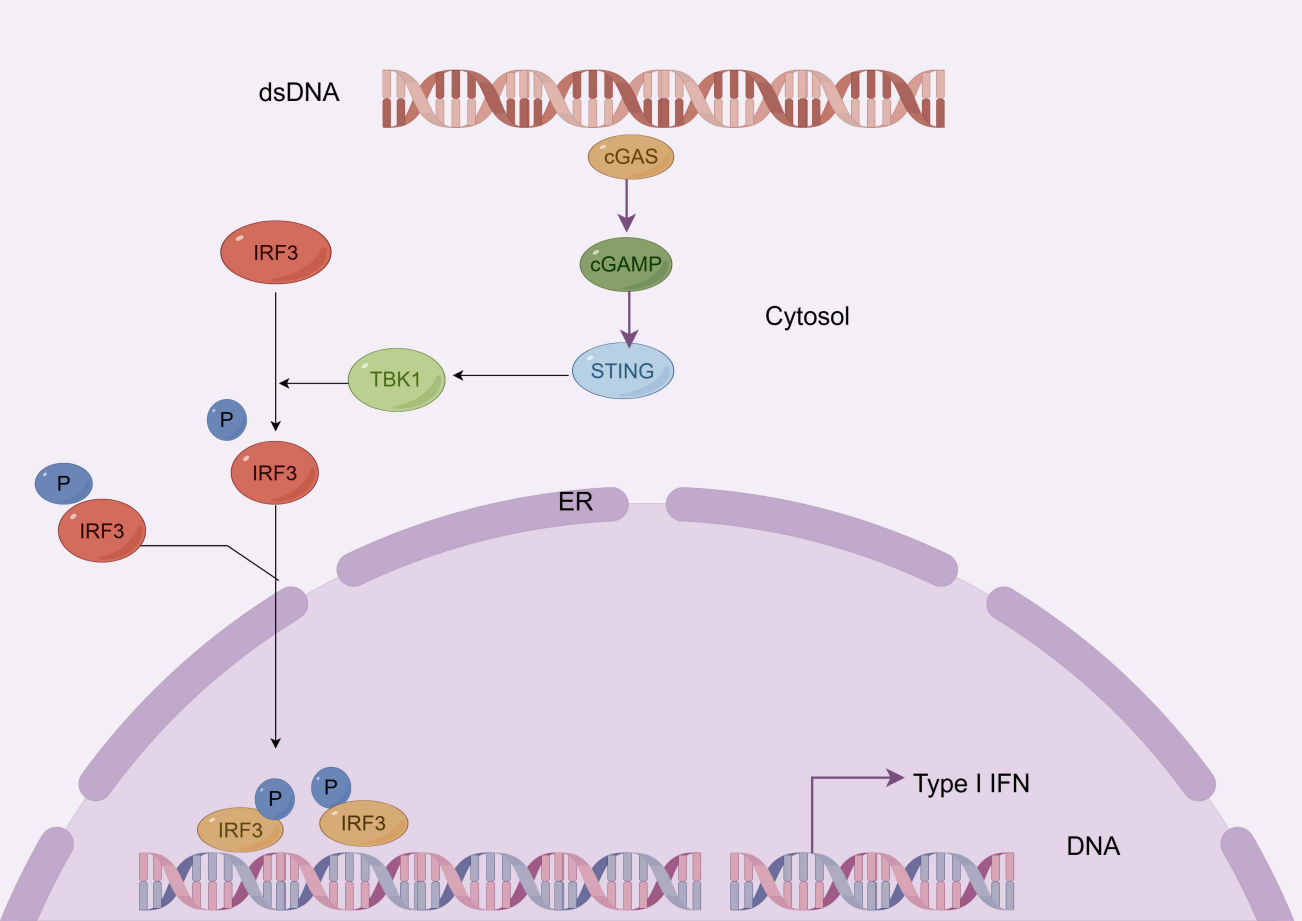
The mechanism of stimulator of interferon genes.

The design concept of immunostimulatory ADCs is to deliver immune agonists, such as Toll-like receptor 7/8 (TLR7/8) agonists or stimulator of interferon genes (STING) agonists, to the tumor microenvironment via antibodies, thus avoiding the toxicity issues that may arise from systemic administration of traditional immune agonists (121) (**Figure 3**).

In immunostimulatory ADCs, TLR7/8 and STING agonists enhance innate immune responses, thereby promoting adaptive immune responses (122). TLR7 and TLR8, as conserved innate immune molecules, play a significant role in the tumor microenvironment (121). They activate NF-κB through MyD88-dependent signaling pathways and promote the secretion of various cytokines and chemokines, such as interleukin-6 (IL-6), tumor necrosis factor-α (TNF-α), interleukin-12 (IL-12), and CXC motif ligands 9 and 10 (CXCL9, CXCL10), which enhance the infiltration and activation of antitumor lymphocytes (123). Concurrently, STING agonists initiate type I interferon signaling pathways, further enhancing T cell adaptive immune responses and bolstering the immune system’s ability to recognize and attack tumors (124).

Immunostimulatory ADCs typically combine TLR7/8 and STING agonists to synergistically enhance immune effects (125). For instance, nanovaccines based on these two agonists have been shown to elicit a broader cytokine response, such as interferon-γ (IFN-γ), IL-2, and interleukin-12, which further strengthens the anticancer effect (126, 127). This combinatorial strategy not only reshapes the tumor microenvironment but also improves the response rate to cancer therapy, helping to address some of the challenges faced by traditional ADC drugs.

Immunostimulatory ADCs can convert “cold tumors” into “hot tumors” that are sensitive to immunotherapy by activating the innate immune system to enhance adaptive immune responses, thereby improving antitumor effects (128). The latest preclinical studies have shown that immunostimulatory ADCs can effectively promote immune cell infiltration in various tumor models and increase the tumor’s response to immune checkpoint inhibitors (129). However, these drugs also face challenges, such as the potential for severe side effects due to systemic nonspecific immune responses (129). Therefore, optimizing the design of immunostimulatory ADCs and the selection of linkers has become one of the important directions in current research.

Additionally, the combination of immunostimulatory ADCs with other immunotherapies is actively being explored. For example, the combination of immunostimulatory ADCs with immune checkpoint inhibitors (ICIs) may further enhance antitumor activity, especially in solid tumors. Recent research has focused on identifying new checkpoint targets, such as TIGIT, TIM-3, and LAG-3, to overcome resistance mechanisms faced by current therapies (130). Researchers hope to improve treatment outcomes by developing mAbs targeting these new targets, providing new treatment options for cancer patients.

The combination of ICIs with chemotherapy, targeted therapy, and anti-angiogenic drugs has shown significant efficacy in various solid tumors (131). For instance, in clinical trials, the combination of PD-1 inhibitors with anti-angiogenic agents, radiotherapy, or chemotherapy has improved treatment outcomes (131). This combination therapy not only increases overall survival and objective response rates but also demonstrates potential synergistic effects in certain tumor types.

Although immunostimulatory ADCs are still in the early stages of development and have not yet received FDA approval, they have made positive progress in preclinical studies, with several companies are advancing related drugs into clinical trials (132, 133). With a deeper understanding of the mechanisms of immunostimulatory ADCs and advancements in technology, these drugs are expected to play a significant role in cancer treatment.

#### 5.2.4 Degradative-antibody conjugates

**Figure 4:**
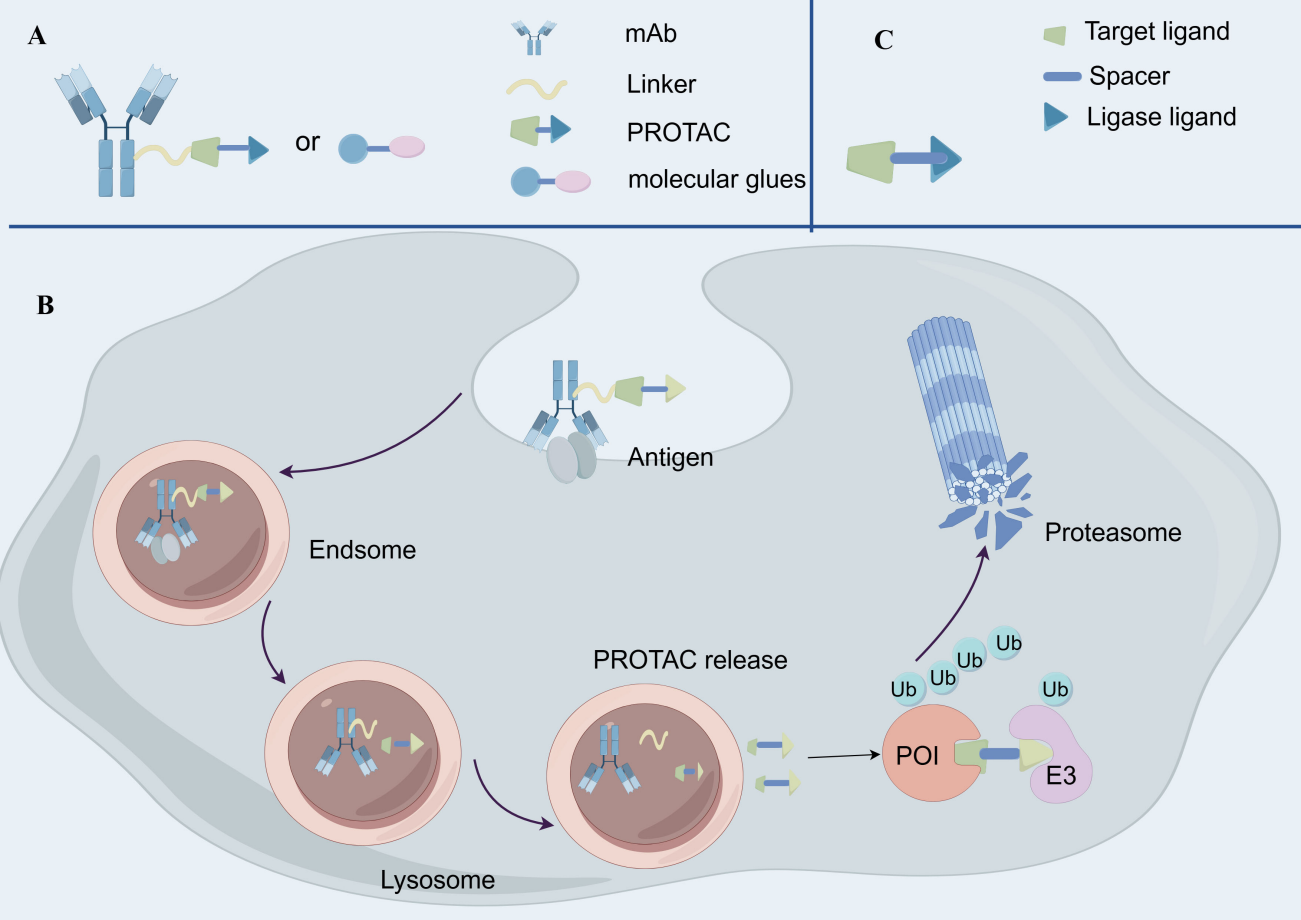
The structure and mechanism of Degradative-Antibody conjugate. **(A)** The structure of Degradative-Antibody conjugate. **(B)** the mechanism of action of Degradative-Antibody conjugate. **(C)** The structure of Proteolysis Targeting Chimeras.

Degradative-Antibody conjugates (DACs) represent an emerging drug delivery strategy that combines the advantages of ADCs and protein degraders, aiming to target and degrade specific disease-associated proteins (134) (**Figure 4**). DACs hold significant potential for application in cancer therapy.

The fundamental principle of DACs involves mAbs that first recognize and bind to tumor-associated antigens, subsequently delivering degradative agent molecules into the target cells (135). Intracellularly, the linker is cleaved under specific conditions to release the degradative agent molecules, such as proteolysis-targeting chimeras (PROTACs) or molecular glues (135) (**Figure 4**). These degradative agents induce ubiquitination of the target proteins via E3 ubiquitin ligases, leading to proteasomal degradation. Unlike traditional ADCs, DACs utilize protein degraders as payloads instead of cytotoxic drugs (74). Consequently, DACs can more precisely target specific proteins, reducing toxic effects on normal cells. Moreover, protein degraders typically have a lower bioavailability requirement, allowing DACs to deliver these molecules *in vivo* without relying on high bioavailability (136).

In recent years, researchers have developed various targeted intracellular protein degraders, including Proteolysis Targeting Chimeras (PROTACs), Proteolysis Targeting Antibodies (PROTABs), and Lysosome Targeting Chimeras (LYTACs) (137, 138). These technologies provide novel strategies for the degradation of cell surface and membrane proteins, expanding the therapeutic potential beyond traditional small molecule inhibitors and mAbs. While DACs primarily focus on delivering cytotoxic payloads to cancer cells, these protein degradation technologies complement DACs by offering alternative mechanisms to eliminate target proteins, particularly those that are undruggable or resistant to conventional therapies (135). For instance, PROTACs and PROTABs leverage the ubiquitin proteasome system to degrade intracellular proteins, while LYTACs exploit the endolysosomal pathway to degrade extracellular and membrane proteins (137, 138). This complementary approach broadens the scope of therapeutic strategies for cancer treatment, addressing targets that may not be effectively targeted by DACs alone.

In summary, antibody-directed protein degraders represent a groundbreaking technology with the potential to offer unique therapeutic options for cancer patients. However, most of these drugs are still in the early preclinical development stages and require further medicinal chemistry research and preclinical evaluation to ensure their safety and efficacy (139).

#### 5.2.5 Dual-payload ADCs

Most solid tumors are composed of heterogeneous cancer cell subpopulations, each with distinct gene expression profiles and varying sensitivities to drugs with different mechanisms of action (140). Consequently, combination therapies involving multiple drugs with diverse modes of action are frequently utilized in clinical practice. Dual-Payload ADCs hold the potential to act as single agents, capable of eliciting additive or synergistic effects, and overcoming drug resistance in patients with treatment-refractory tumors while offering a straightforward dosing regimen (140, 141).

In 2017, a method for producing Dual-payload ADCs using branched chemical linkers containing two orthogonally masked cysteine residues was reported (141). This innovative approach enabled the homogeneous coupling of both MMAE and monomethyl auristatin F (MMAF) to anti-CD30 antibodies at a DAR of 16 (142, 143). The Dual-Payload ADCs demonstrated potent activity in a mouse xenograft model of mesenchymal large cell lymphoma (ALCL) expressing CD30+MDRn (141). This dual payload strategy has the potential to act as a single agent, triggering additive or synergistic effects, and overcoming drug resistance in patients with treatment-refractory tumors while maintaining a straightforward dosing regimen.

Dual-Payload ADCs have been further explored for their clinical potential as they combine two different payload classes, moving beyond ADCs loaded with cytotoxic agents such as MMAE and MMAF. For instance, the combination of asterlin with TLR agonists conjugated to anti-FolRα antibodies has demonstrated synergistic anti-tumor activity and immune memory in mouse models (141, 144).

However, not all studies testing Dual-Payload ADCs have demonstrated meaningful synergistic effects, particularly those involving different payload classes (145–147). For example, investigators developed an anti-HER2 ADCs equipped with MMAE plus SG3457, an ultrapotent PBD dimer capable of damaging DNA via crosslinking (145). Likewise, a HER2-targeted ADCs equipped with MMAF and the highly potent topoisomerase II inhibitor PNU-15968 was developed (146). Despite both of these ADCs being capable of exerting dual mechanisms of action, neither agent demonstrated an improvement *in vitro* potency when compared with their corresponding single-drug ADCs.

These findings highlight the importance of selecting payloads with appropriate mechanisms of action, ensuring balanced potency between the two payloads selected and optimizing DARs to achieve optimal therapeutic outcomes. Efforts to fully understand and maximize the potential of Dual-Payload ADCs are still in the early stages of exploration.

## 6 Conclusion

ADCs have emerged as a transformative therapeutic modality in oncology, combining the precision of mAbs with the potency of payload to deliver targeted therapy. Over the past decade, significant advancements in ADCs design, conjugation technologies, and payload selection have driven their clinical success and commercialization (38, 148).

The structural optimization of ADCs, including advancements in antibody engineering, linker chemistry, and payload design, has been pivotal in enhancing their therapeutic index. Site-specific conjugation technologies have improved homogeneity and stability, while innovative payloads, such as immunomodulators and protein degraders, have expanded the therapeutic potential of ADCs. Novel ADCs designs, including bispecific ADCs, immunostimulatory ADCs, DACs, and Dual-Payload ADCs, are addressing challenges such as tumor heterogeneity, drug resistance, and off-target toxicity (5). These next-generation ADCs hold promise for overcoming limitations of traditional therapies and improving outcomes in refractory cancers.

Despite these advancements, ADCs continue to face significant challenges in clinical translation. Issues such as complex pharmacokinetics, payload release control, and the emergence of resistance mechanisms necessitate further innovation (149). The development of spatially and temporally controlled release mechanisms, improved linker stability, and strategies to mitigate antigen heterogeneity are critical areas of research. Additionally, the high manufacturing costs and technical hurdles associated with ADCs production require optimization to ensure broader accessibility and affordability.

Looking ahead, the integration of ADCs with emerging therapeutic modalities, such as immune checkpoint inhibitors and targeted protein degraders, is expected to enhance their efficacy and overcome resistance. The continued evolution of ADCs technologies, coupled with advances in companion diagnostics and precision medicine, positions ADCs as a cornerstone of next-generation cancer therapy. As the field progresses, ADCs are poised to play an increasingly important role in the treatment of heterogeneous and refractory cancers, offering new hope for patients with limited therapeutic options.
